# Guidelines on the performance evaluation of motif recognition methods in bioinformatics

**DOI:** 10.3389/fgene.2023.1135320

**Published:** 2023-02-07

**Authors:** Igor V. Deyneko

**Affiliations:** Laboratory of Functional Genomics, К.А. Timiryazev Institute of Plant Physiology RAS, Moscow, Russia

**Keywords:** cis-regulatory modules, DNA motif detection, enhancers, promoters, DNA sequence analysis, gene regulation

## 1 Introduction

The accurate discovery of DNA and RNA regulatory motifs and their combinations is still a topic of active research, focusing to date mainly on the analysis of ChIP-Seq data (Kingsley et al., 2019; Kong et al., 2020), on gene co-expression analysis (Rouault et al., 2014; Teague et al., 2021) and on the general investigation of the properties of binding motifs (Zeitlinger 2020). Many bioinformatics methods have been (Zambelli et al., 2013) and are still being developed (Bentsen et al., 2022; Hammelman et al., 2022) to improve prediction accuracy and fully address the advantages of novel experimental and computational techniques, such as that based on deep learning (Auslander et al., 2021).

However, when it comes to the practical use of bioinformatic predictors, a researcher is often puzzled, first by selecting an appropriate bioinformatic program and then by a huge list of predictions that such programs usually produce. Once several programs are used to increase the chances of one at least finding a real functional motif, the list of predictions becomes too long for experimental verification (Deyneko et al., 2016), even though independently found similar motifs are more likely to be correct and can be given higher priority (Machanick and Kibet, 2017).

The main problem that complicates the choice of a favorable approach for a specific task is the insufficient number of comparative tests of the published methods, partly due to the difficulty of defining a universal motif assessment approach (Kibet and Machanick, 2016). The inadequate testing of many newly suggested algorithms has already been discussed (Smith et al., 2013) and can be summarized as 1) an insufficient and subjective selection of methods for comparison; 2) use of non-common metrics; and 3) use of non-standard datasets.

Nevertheless, many studies that present novel methods for motif detection repeatedly appear without adequate comparative evaluation. The main issues include comparison against no or only a single method, despite several comparable methods existing (Alvarez-Gonzalez and Erill, 2021; Hammelman et al., 2022), the use of only one dataset, usually with unknown true positives (Levitsky et al., 2022), and the use of uncommon statistical metrics (Zhang et al., 2019). The last can be exemplified with a criterion of the correct prediction—if, within the top ten, there is a motif similar (not identical!) to the original, the motif is counted as positively recovered. In real applications, when the target motif is unknown, the reliability of such predictions is far from being experimentally testable. In contrast, there are many methods with well-performed comparisons, including novel deep learning methods (Bentsen et al., 2022; Iqbal et al., 2022).

This work is addressed not only to researchers, who may use the presented principles to better reveal the power of the software presented, but also to peer reviewers and journal editorial boards, who may use it as a starting point for their own requirements for software articles. Obviously, comprehensive comparative testing of new methods will not only reveal the best fields of application but, most importantly, will help wet-lab researchers to navigate through bioinformatics topics.

## 2 Guidelines on comparative testing

**FIGURE 1:**
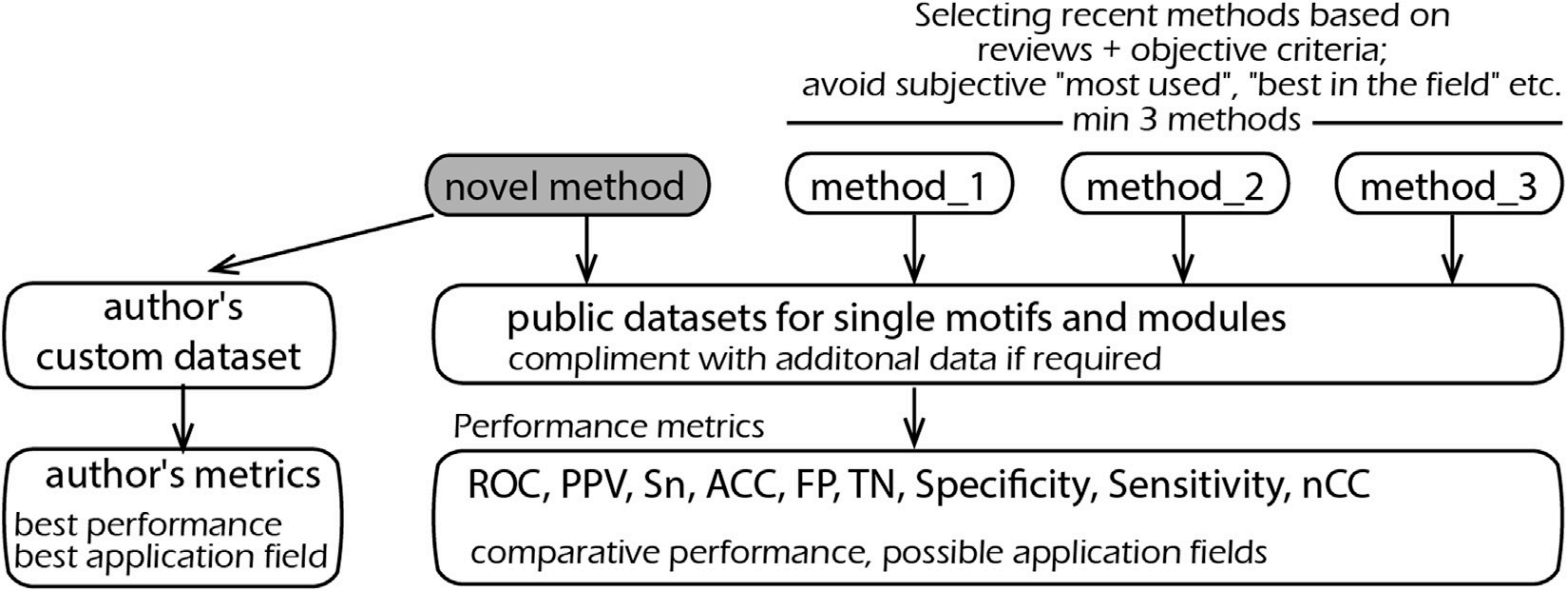
Schematic representation of the proposed principles on the selection of methods, metrics, and datasets for comparative testing.

Overall, the situation can be improved by introducing the following three principles, schematically represented in Figure 1.

### 2.1 Selecting methods for comparison

There must be a clear logic as to why specific methods have been selected for benchmarking; any subjective choice of certain programs should be avoided. The easiest and most objective way is to use a review article. The classical examples are the works of Sandve et al. (2007) and Klepper et al. (2008), which additionally provide an online system for methods comparison. Other reviews worth noting are Tran and Huang (2014) and Jayaram et al. (2016).

Methods based on novel computational principles and/or experimental data are always welcome, provided that their performance is also properly evaluated against “old methods.” If, by some modification of an input (output), such methods can be adapted for testing, this should be carried out and the methods included in the comparative list. Notwithstanding, the gold standard for comparisons should comprise three methods and preferably five—always preferring the most recent.

### 2.2 Selecting datasets

In its basic definition, DNA motif detection is a well-defined problem about a dataset of nucleotide sequences—either long as promoters or short as ChIP-seq segments. Therefore, it should (almost) always be possible to run a new program on existing data, and there are many such examples (Klepper et al., 2008; Deyneko et al., 2013). Thus, the use of common and publicly available datasets should be obligatory. Once a new algorithm requires additional information, such as expression values, genome positioning, and conservation, standard datasets can be complemented with reasonable values required for a correct comparison. This will reveal how a new method works on “old data,” ensure a fair testing against other methods, and, most importantly, demonstrates the added value of this additional information. For example, if gene expression values are required, a sequence-only dataset can be modified by assigning “1s” to foreground and “0s” to background sequences. This will clearly show the performance gain with respect to the use of such additional information.

The use of self-made datasets can only be accepted as complimentary to standard ones. Even if a method is developed to address a particular problem and does not operate promisingly on standard datasets, the results should still be presented. This will clearly show where a method outperforms other methods and on which data it does not, so that an application niche is clearly defined. Authors should not be afraid of a possibly very narrow application field for their research. Instead, a clear definition will help practitioners find and use the appropriate program before they give up in disappointment after a series of unsatisfactory attempts.

In implementing new methods, researchers should also be cautious about integrating multiple steps into one executable. It is certainly very convenient to analyze the raw data in one go, but this will greatly reduce the field of application. For example, giving human gene names as input, instead of promoter sequences, makes it impossible to analyze the genes of mice, plants, or bacteria. Extracting specific genomic regions is today a trivial task, although it may be implemented as an option for convenience.

### 2.3 Selecting performance metrics

Methods including ROC curves, false-positives, true-negatives, selectivity and sensitivity, nucleotide correlation coefficients, and positive predictive values are to be used as metrics (Vihinen 2012; Jayaram et al., 2016). If a novel method or dataset does not allow standard metrics, others may be used, provided that it is clearly explained why standard metrics are not applicable. One should avoid giving subjective assertions of performance like “Identified all 40 conserved modules reported previously” without mentioning how many other modules (false positives) were also identified, or referring to the literature as the only measure of correct predictions (El-Kurdi et al., 2020). Reference to the literature is fully valid and useful, provided that comprehensive statistics are given. It is notable that statistical measures like *p*-values are often method-specific—they depend on a method’s internal calculations. So, the *p*-values of different methods should be compared with caution.

## 3 Good practices in comparative testing

As examples of thorough comparative testing, two programs will be discussed—MatrixCatch for recognizing cis-regulatory modules (Deyneko et al., 2013) and a predictor of acetylcytidine sites in mRNA based on novel deep learning methodology (Iqbal et al., 2022).

MatrixCatch uses a database of known composite modules as the basis for recognition. Three classes of comparisons were performed: with methods based on the same principle, with statistical methods, and on the recognition of cis-modules on a real dataset. Next, we briefly discuss the three classes of comparative testing and how they align with the suggested guidelines.

At the time of developing MatrixCatch, two other methods—based on the same principle of using known examples of composite modules—were available. They were compared against the same sequence dataset, with ROC curves as a performance characteristic.

The second type of comparison was performed against statistical methods for motif detection. The difference from the previous comparison is that the motifs and modules are found solely by nucleotide frequency statistics. For such “*de novo*" modules, there is no experimental (or any other) evidence for their functionality. The advantage of such methods is their ability to find truly new motifs and modules. In contrast, MatrixCatch uses a library of experimentally verified modules and is therefore limited to its known repertoire. Although these two types of method use different principles, it is important from a practical point of view to know which method(s) provides the best chance of finding real motif(s) and explain, for example, a co-regulation of genes in an RNA-seq experiment. The tests were performed according to the benchmarking presented in a review by Klepper et al. (2008), which includes six datasets of DNA sequences, nine methods, and several performance characteristics common to all methods (methods based on reviews—Figure 1).

Finally, testing was illustrated by the detection of cis-modules on 11 sets of tissue-specific promoters (authors’ custom data—Figure 1). Regulatory elements presumably existing in promoters are unknown, and therefore, measuring such factors as false positives, ROC, or otherwise cannot be calculated. The performance was measured as the specificity of the best module and equal to the ratio of the number of promoters with recognized cis-module in a positive set to the respective number in the negative set (authors’ custom metrics—Figure 1). Such a definition is the most indicative in real applications, where a researcher seeks to identify elements that occur preferentially in the dataset of interest. Moreover, this measure can be applied to all recognition methods despite their different search logics and output formats.

Another example is a method for recognition of N4-acetylcytidine sites in mRNA (Iqbal et al., 2022) based on novel deep learning methodology. The method was tested on publicly available reference data; ROC, precision–recall curves, and accuracy, specificity, and sensitivity measures were used to evaluate the consistency of classification. An interesting point is that the method was compared to the three “old-style” machine learning methods, including regression and support vector machine. This not only serves as a bridge between new and conventional methods but also shows its advantages over, for example, regression analysis available in most statistical software.

## 4 Conclusion

The comprehensive testing of novel methods seems to be as laborious as the developing methods themselves and thus requires longer result sections in manuscripts. Publishing an “application note” or similar with an imposed page limit forces authors to search for a specific dataset or simulation settings for which their method works better than existing ones. This leads to a very subjective presentation and over-optimism in bioinformatics research—and disillusion in practice (Boulesteix 2010). Following the aforementioned guidelines will simplify and unify methods benchmarking designs and will reveal their best application fields. Establishing similar practical recommendations in other subfields of bioinformatics will facilitate application by practitioners and true innovation by bioinformaticians.
